# Half-Heusler phase TmNiSb under pressure: intrinsic phase separation, thermoelectric performance and structural transition

**DOI:** 10.1038/s41598-023-28110-4

**Published:** 2023-01-28

**Authors:** Kamil Ciesielski, Karol Synoradzki, Damian Szymański, Kazuki Tobita, Katarzyna Berent, Patryk Obstarczyk, Kaoru Kimura, Dariusz Kaczorowski

**Affiliations:** 1 Institute of Low Temperature and Structure Research, Polish Academy of Sciences, ul. Okólna 2, 50-420 Wrocław, Poland; 2 Institute of Molecular Physics, Polish Academy of Sciences, M. Smoluchowskiego 17, 60-179 Poznań, Poland; 3 Department of Advanced Materials Science, The University of Tokyo, 5-1-5 Kashiwanoha, Kashiwa, Chiba 277-8561 Japan; 4 AGH University of Science and Technology, Academic Centre for Materials and Nanotechnology, al. Mickiewicza 30, 30-059 Kraków, Poland; 5 Centre for Advanced Materials and Smart Structures, Polish Academy of Sciences, 50-422 Wrocław, Poland

**Keywords:** Electronic properties and materials, Semiconductors, Materials science

## Abstract

Half-Heusler (HH) phase TmNiSb was obtained by arc-melting combined with high-pressure high-temperature sintering in conditions: *p* = 5.5 GPa, $T_{HPHT}$ = 20, 250, 500, 750, and 1000 $^{\circ }$C. Within pressing temperatures 20–750 $^{\circ }$C the samples maintained HH structure, however, we observed intrinsic phase separation. The material divided into three phases: stoichiometric TmNiSb, nickel-deficient phase TmNi$_{1-x}$Sb, and thulium-rich phase Tm(NiSb)$_{1-y}$. For TmNiSb sample sintered at 1000 $^{\circ }$C, we report structural transition to LiGaGe-type structure (*P*$6_3$*mc*, *a* = 4.367(3) Å, *c* = 7.138(7) Å). Interpretation of the transition is supported by X-ray powder diffraction, electron back-scattered diffraction, ab-initio calculations of Gibbs energy and phonon dispersion relations. Electrical resistivity measured for HH samples with phase separation shown non-degenerate behavior. Obtained energy gaps for HH samples were narrow ($\le$ 260 meV), while the average hole effective masses in range 0.8–2.5$m_e$. TmNiSb sample pressed at 750 $^{\circ }$C achieved the biggest power factor among the series, 13 $\upmu$WK$^{-2}$cm$^{-1}$, which proves that the intrinsic phase separation is not detrimental for the electronic transport.

## Introduction

Prolific interplay of physics and chemistry inherent to the field of thermoelectrics has been gathering attention of the scientific community throughout the decades. Among many interesting groups of materials considered for this purpose, half-Heusler (HH) phases seem to be closest to real-life application, due to the fact that high energy conversion efficiency^1^ can be hither supplemented by relatively low price of constituent elements, good mechanical properties, as well as decent thermal stability^2^. Apart of thermoelectricity, HH compounds are also investigated from multiple different perspectives, e.g. magnetocaloric applications^3^, superconductivity coexisting with antiferromagnetism^4^, heavy fermion behavior^5^, half-metallic ferromagnetism^6^, and anomalous structural transitions^7^. Recently *f*-electron systems gathered special attention due to their nontrivial topological properties^8^.

**Figure 1:**
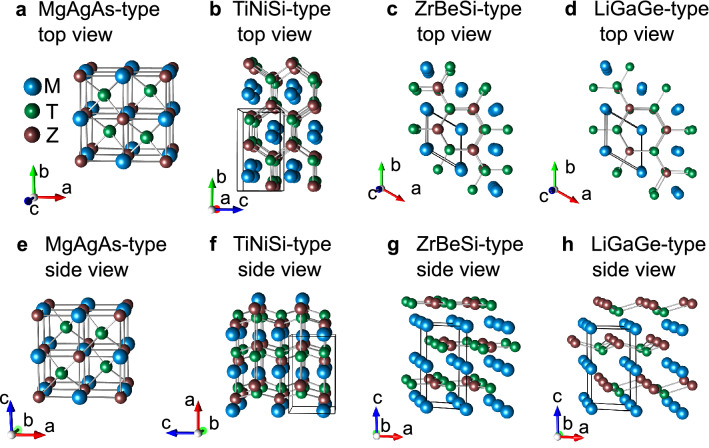
Different types of crystal structures attained by equiatomic ternaries with general composition *MTZ*, see text for details. Figure prepared in Vesta^16^ software.

General chemical composition of HH phases is *MTZ*, where *M* denotes for early transition metal or rare-earth (*RE*) element, *T* stands for late transition metal, and *Z* is *p*-electron element. Structural prototype of HH phases is MgAgAs, space group being $F{\bar{4}}3m$ (no. 216). The unit cell is depicted in Fig. 1a, while the atomic coordinates are gathered in Supplementary Table S1. The structure can be described as rock-salt lattice of *M*-*Z* atoms, with half of the voids in small *M-Z* cubes filled with *T* elements. The second half of 8-coordinated voids are 4*d* ($\frac{3}{4}\frac{3}{4}\frac{3}{4}$) Wyckoff position, whose in ideal structure remain unoccupied. Despite apparent simplicity of the unit cell, HH compounds are known to comprise bounty of structural peculiarities. The materials based on *d*-electron elements, e.g. ZrNiSn, are known for their low energy interstitial defects on nominally empty 4*d* site, and Zr-Sn antisites^9,10^. The disorder was found to be beneficial for thermoelectric energy conversion^11,12^. The rare-earth bearing counterparts are known to exhibit different type of dominant defects. Depending on method of synthesis they are known to comprise either vacancies on late transition metal site 4*c* ($\frac{1}{4}\frac{1}{4}\frac{1}{4}$), e.g. nickel in ScNiSb^13^, or split positions of 4*c* slot to quarterly occupied position 16*e*(*x*,*x*,*x*) with *x* = 0.256-0.260^14,15^.

Furthermore, selected HH compounds show phase separation (PS), i.e. their polycrystalline samples have regions strongly differing in composition, while maintaining the MgAgAs-type structure. PS was reported before for materials with general composition Ti$_{1-x-y}$Zr$_x$Hf$_y$*MZ* where *M* = Co, Ni, and *Z* = Sb, Sn^17–21^. The structural arrangement of samples was usually division into (Zr, Hf)-rich dendritic phase, and interdendritic grains enriched in titanium. The phenomenon is assumed to origin from different atomic radii of early transition metal atoms ($r_{Ti}$ = 2.11 Å, $r_{Zr, Hf}$ $\approx$ 2.23 Å) and significantly distinct temperature of solidification: 1453 K for TiNiSn, 1708 K for ZrNiSn and 1760 K for HfNiSn^22^. In the case of *d*-electron HH compounds, PS was found to be beneficial for thermoelectric energy conversion due to enhanced phonon scattering with simultaneously small effect on electrical properties^17–20,23^. The HH samples maintain peculiar PS microstructure after extensive temperature cycling^24^, which is critical from perspective of future application. The phenomenon received sizable attention of theoretical part in thermoelectric community, see e.g. Refs.^25–27^. PS was found to be important also for other groups of TE materials: GeTe^28–30^, PbTe^31,32^, AgPb$_m$SbTe$_{m+2}$^33,34^ and SnSe^35^.

At elevated temperatures and/or high pressures HH compounds undergo interesting phase transitions. TiPtGe was reported to transform at 885 $^{\circ }$C from cubic MgAgAs-type structure to orthorhombic TiNiSi-type cell with large volume contraction of 10%, despite increase in temperature^7^. Similar type of phase transition was observed for YbPdSb^36^. In the TiNiSi-type structure, Ni and Si form 6-atoms puckered rings, while Ti forming zig-zag chains along [100] direction, filling the Ni-Si tunnels. The reader is referred to Fig. 1b, and Fig. 1f for the TiNiSi-type unit cell in top and side view, respectively. The related atomic positions are gathered in Supplementary Table S1. On the path from MgAgAs-type to TiNiSi-type transformation, the authors of Ref.^7^ suggested presence of metastable ZrBeSi-type structure, space group *P*63/*mc*. Experimentally, ZrBeSi-like structure was stabilized in HH phases VCoSb and VFeSb, crystallizing in MgAgAs-type structure at atmospheric pressure, while hexagonal unit cell was observed after synthesis in extreme conditions: 5.0 GPa and 900 $^{\circ }$C^37,38^. Similarly to TiNiSi-type structure, ZrBeSi-type cell also hosts form 6-atoms rings, here made from Be and Si atoms. The reader is referred to Fig. 1c for top view of the structure and Fig. 1g for the side view. Interestingly, the so-formed Be-Si planes are here spatially separated from Zr layers, so the crystal structure might be considered as quasi 2-dimensional, c.f. Fig. 1g. ZrBeSi-type structure is sometimes also referred as ordered version of Ni$_2$In-type cell, or AlB$_2$-type. The latter is in principle binary subcell of ZrBeSi. See Ref.^39^ for exhaustive description of these crystal structures. In the series *RE*NiSb, materials with light rare-earth elements *RE* = La, Ce, Pr, Nd, Sm crystallize with hexagonal ZrBeSi-type structure, while heavier counterparts with *RE* = Tb, Dy, Ho, Er, Tm, Lu, attain HH structure^40^. GdNiSb being in the middle between those two groups can be obtained in both polymorphs^40^. Last, but not the least CaAuBi^41,42^ and YbAuBi^43^ crystallize in MgAgAs-type and LiGaGe-type (sg. *P*63*mc*), see Fig. 1d, h. The latter prototype can be viewed as distorted version of ZrBeSi-type cell. It attains majority of features characteristic for ZrBeSi-type structure, but all atomic positions have relaxed *z* coordinates, which leads to puckering of Ga-Ge rings; see Fig. 1h for the side view.

Recently, thermoelectric figure of merit *ZT* = 1.5 ($ZT=\frac{S^2}{\rho \kappa }T$, where *S* denotes thermopower, $\rho$ stands for electrical resistivity, while $\kappa$ is thermal conductivity) at elevated temperatures was reached for several HH compositions: TaFeSb-^44^, NbFeSb-^45^, and ZrCoBi-based materials^46^. Good thermoelectric performance of HH compounds results from values of the power factor (*PF* = $S^{2}/\rho$) as high as 50 $\upmu$WK$^{-2}$cm$^{-1}$. Relatively large thermal conductivity ($\kappa \ge$ 3 WK$^{-1}$m$^{-1}$), however, is still an obstacle to overcome. Preliminary research shown that *f*-electron HH phases can exhibit lower $\kappa$ than the *d*-electron counterparts, where pristine compounds are compared: thermal conductivities near room temperature for exemplary rare-earth (*RE*) based HH phases were e.g. 5 WK$^{-1}$m$^{-1}$ for HoPdSb^47^ and in 3-4 WK$^{-1}$m$^{-1}$ range for TmNiSb^48,49^, while for *d*-electron compounds $\kappa$ at 300 K was 9 WK$^{-1}$m$^{-1}$ for ZrCoBi^46^, 13 WK$^{-1}$m$^{-1}$ for FeVSb^47^, 6 WK$^{-1}$m$^{-1}$ for HfNiSn, and 8 WK$^{-1}$m$^{-1}$ for ZrNiSn^50^. Suppressed phonon transport in *RE*-containing materials results from their lower Debye temperatures, high density and sizable intrinsic atomic disorder^51^.

TmNiSb is representative of rare-earth bearing HH compounds^13–15,49^ crystallizing with lattice parameter *a* = 6.241 Å, as reported by Dwight et al.^52^ and 6.225 Å by Pecharski and coworkers^53^. Sportouch et al. investigated its thermoelectric properties below room temperature, which resulted in the lowest thermal conductivity at 300 K among other *RE*NiSb (3 WK$^{-1}$m$^{-1}$), and *PF* = 1 $\upmu$WK$^{-2}$cm$^{-1}$^48^. Our reports on thermoelectric properties of *RE*NiSb compounds in wider temperature range confirmed low thermal conductivity of TmNiSb^49,51^ and shown that at around 700 K the compound can attain high power factor, depending on method of synthesis: 11 $\upmu$WK$^{-2}$cm$^{-1}$ for arc-melting sample^49^, and 17 $\upmu$WK$^{-2}$cm$^{-1}$ for specimen synthesized by combination of arc-melting and spark plasma sintering^49^.

Other interesting method for preparation of thermoelectric materials is high-pressure high-temperature sintering (HPHT), which employs rapid compression of elemental constituent in pressures of several GPa and elevated temperatures^54^. Up to date the technique has been used for improving thermoelectric performance of PbTe^54^, Bi$_2$Te$_3$^55^, skutterudites^56^, and SnSe^57^. Main influence of HPHT process on thermoelectric transport is enhanced phonon scattering due to lattice defects introduced on different size scales^58^. In our previous work, we studied thermoelectric properties of Sc$_{1-x}$Tm$_x$NiSb half-Heusler phases. Formation of solid solution lead to decrease of thermal conductivity in the alloy space with respect to parent compounds and the best performance was achieved for Sc$_{0.75}$Tm$_{0.25}$NiSb (*PF* = 12 $\upmu$WK$^{-2}$cm$^{-1}$ at 630 K)^59^. This study, however, was not focused on optimization of synthesis conditions; the samples were pressed uniformly in 5.5 GPa and 750 $^{\circ }$C.

**Figure 2:**
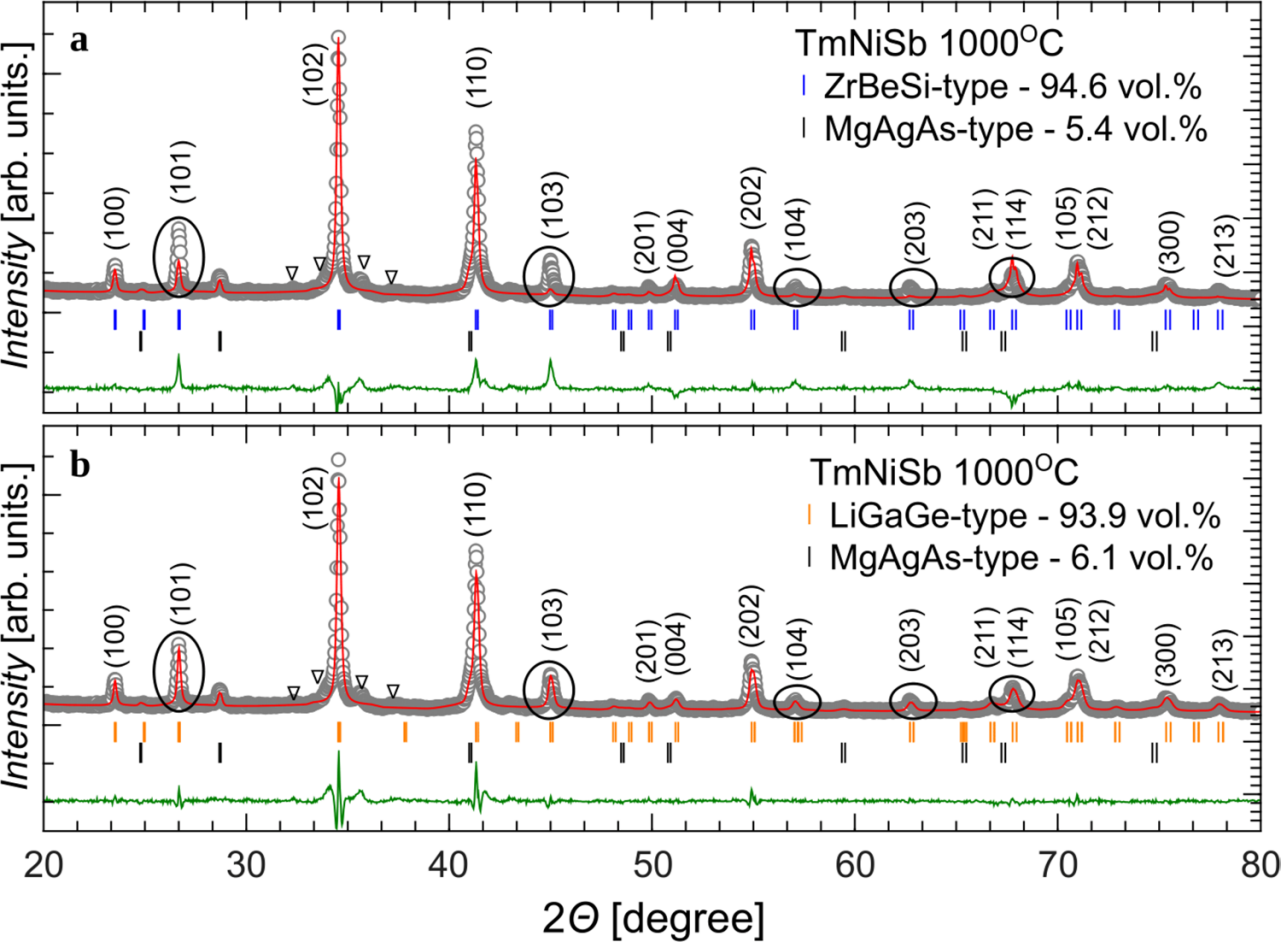
(**a**) XRD patterns of TmNiSb specimen prepared at 1000 $^{\circ }$C with Rietveld fit using ZrBeSi-type and MgAgAs-type phases. Panel (**b**) present Rietveld fit for the same sample with LiGaGe-type and MgAgAs-type phases. Black ovals mark Bragg maxima for which the most significant differences were noticed between the two refinements, while triangles denote Bragg peaks of impurity phase. Miller indices are given for the most pronounced peaks of ZrBeSi-type and LiGaGe-type phases.

Inspired by the previous reports on HPHT synthesis and curious about behavior of *RE* HH compounds under pressure, we explore influence of different HPHT synthesis conditions on TmNiSb. Extraordinary structural features were noticed depending on temperature of sintering. We observed division of the HH compound through the intrinsic phase separation (PS) into TmNiSb, TmNi$_{1-x}$Sn, and Tm(NiSb)$_{1-y}$ phases. Furthermore, sample prepared at 1000 $^{\circ }$C shown structural phase transition to LiGaGe-type structure. The study is complemented by thermoelectric characterization, which shows that pressure-driven phase separation might be useful for optimization of the performance. This work is part of larger project aimed at investigation of structural, thermoelectric and magnetic properties of rare-earth bearing HH compounds^60–67^.

## Results and discussion

### Powder X-ray diffraction

Powder X-ray diffraction patterns of samples prepared at temperatures 20–750 $^{\circ }$C are displayed in Supplementary Figure S1a. All significant Bragg maxima can be indexed to MgAgAs-type structure. Lattice parameters obtained from Rietveld refinement are: 6.237(1) Å, 6.223(2) Å, 6.244(1) Å, and 6.242(1) Å, for samples prepared at 20, 250, 500, and 750 $^{\circ }$C, respectively. Value of the lattice parameter oscillate closely around those from the previous literature^52^. Bragg peaks in our XRD patterns are rather wide and with slightly irregular profile, see Supplementary Figure S1b for exemplary profile of (422) maximum. This feature might be explained e.g. by large strain and some percentage of nonstoichiometric HH grains with slightly differing lattice parameters. Densities measured by Archimedes method were in 8.5–9.3 g/cm$^3$ range (89–97 % of theoretical single phase density), with the lowest for sample pressed at room temperature and the highest for specimen prepared at 750 $^{\circ }$C. Minuscule impurity peaks marked by asterisks on Supplementary Figure S1a. stem mostly from presence of Ni$_{1-x}$Sb$_x$ solid solutions and small amount of Tm$_2$O$_3$.

For sample synthesized at 1000 $^{\circ }$C diffraction pattern changed significantly. Fig. 2a shows our preliminary fit with two phases: MgAgAs-type and ZrBeSi-type structures, where the latter was assumed by analogy to closely related isostructural analogue GdNiSb^40^. The obtained lattice parameter for HH phase is *a* = 6.22(2) Å, while for ZrBeSi-type *a* = 4.367(3) Å, *c* = 7.139(7) Å. The volume fraction is 5.43(4) % for HH and 94.6(9)% for the hexagonal phase, with global fit quality parameter *GOF* = 3.16. All the atomic coordinates are fixed due to symmetry requirements in these phases; see Supplementary Table S1. Due to visible discrepancies in several maxima intensity (see black ovals in Fig. 2a) we attempted another Rietveld fit with LiGaGe-type phase instead of the ZrBeSi-type. The results are shown in Fig. 2b. Fit quality was visibly enhances, GOF decreased to 1.96. Obtained lattice parameters are virtually equal to those resultant from previous refinement: 6.22(2) Å for HH phase and *a* = 4.366(3) Å, *c* = 7.136(7) Å for LiGaGe-type compound. The refined atomic coordinates allowing puckering of NiSb sheets (see Fig. 1h) are: $z_{Tm}$ = 0.516(2), $z_{Ni}$ = 0.680(1), $z_{Sb}$ = 0.285(1). According to expectations, volume fraction of two respective phases is rather similar to the previous fit: 93.9(7) % for LiGaGe-type phase and 6.1(2) % for HH phase. Experimental density of TmNiSb specimen is 9.76 gm/cm$^3$, i.e. higher than HH samples synthesized at temperatures 20–750 $^{\circ }$C.

We suggest, that maxima marked by triangles in 2$\Theta$ = 32–38 $^{\circ }$ range might correspond to the orthorhombic modification of TmNiSb compound in TiNiSi-type structure. This polymorphic modification was observed previously for other HH compounds^7,36^. However its small relative volume in the currently studied samples combined with low symmetry made the precise structure identification impossible. In future we plan to proceed with differential scanning calorimetry and *in situ* synchrotron diffraction experiment aimed on resolving the plausible second phase transition in TmNiSb.

**Figure 3:**
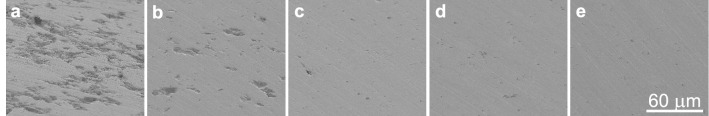
SEM images of TmNiSb samples sintered at temperatures: (**a**) 20 $^{\circ }$C, (**b**) 250 $^{\circ }$C, (**c**) 500 $^{\circ }$C, (**d**) 750 $^{\circ }$C, (**e**) 1000 $^{\circ }$C.

**Figure 4:**
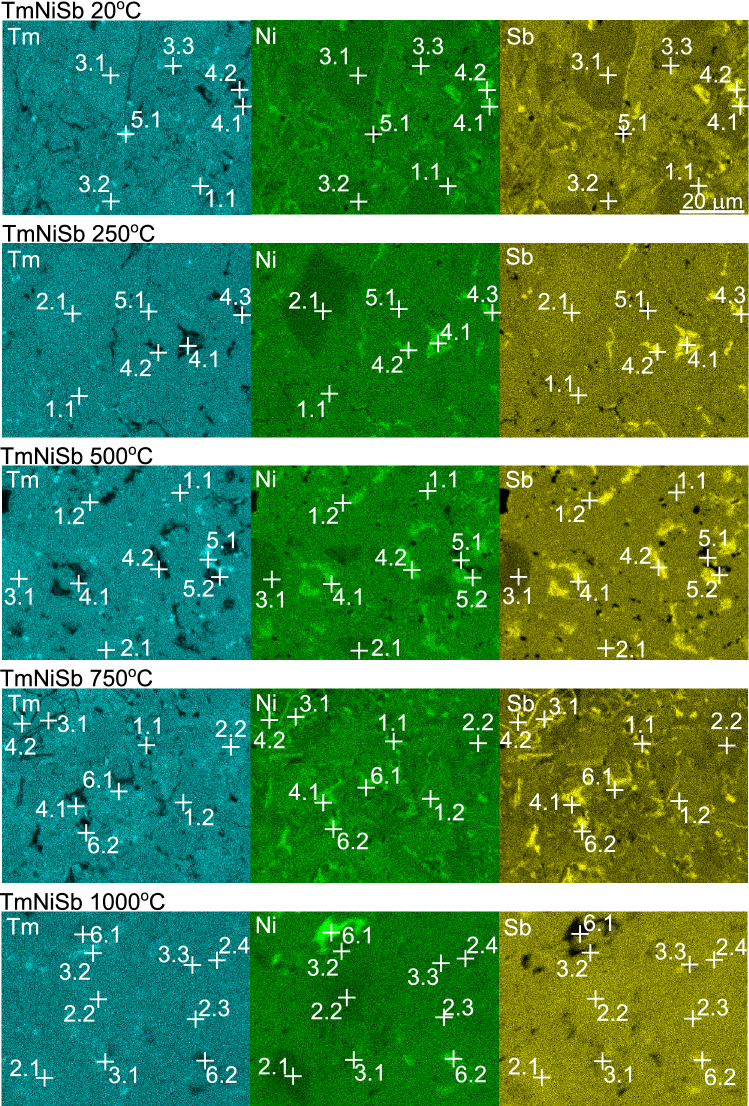
Chemical maps of TmNiSb specimens prepared at temperatures 20–1000 $^{\circ }$C. For results of quantitative analysis see Table 1. Enumeration of point is according to X.Y system, where X denotes captured phase (1—TmNiSb, 2—TmNi$_{1-x}$Sb, 3—Tm(NiSb)$_{1-y}$, 4—NiSb, 5—Tm, 6—Ni rich precipiTtation), and Y is the ordinal number of particular point.

### Scanning electron microscopy, energy dispersive X-ray spectroscopy

SEM images of sample microstructure after sintering under different preforming temperatures are presented in Fig. 3. In order to enhance visibility of morphological features, we tilted the image plate by 70 $^{\circ }$ with respected to the detector. As apparent from the figure, preparation of samples at low temperatures (20 $^{\circ }$C, 250 $^{\circ }$C) results in porous structure. After increasing temperature to 500-1000 $^{\circ }$C, the specimens appear to be more dense, almost indistinguishable from perspective of SEM imaging. Results of energy dispersive X-ray spectroscopy (EDS) analysis are displayed in Fig. 4 and quantified in Table 1. The stoichiometric half-Heusler phase TmNiSb is marked as 1.X on maps, where X is the ordinal number of a point in which EDS analysis was performed. As a result of phase separation there are visible also: non-stoichiometric HH phase depleted in nickel TmNi$_{1-x}$Sb (2.X) and HH phase enriched in thulium Tm(NiSb)$_{1-y}$ (3.X). Assumption of the fact, that all three regions correspond to half-Heusler phases with different types of defects, is justified by rather large amount of defective grains - too large to overlook in pXRD if they corresponded to impurity phases, (c.f. almost phase-pure XRD patterns in Supplementary Figure S1). To confirm that observed hither PS is representative for large areas of TmNiSb samples, we prepared also EDS maps with scale bar 100 $\upmu$m (see Supplementary Figure S2 and corresponding Table S2), which demonstrate that phase distribution is representative for large areas of the samples.

**Table 1:** Results of quantitative EDS analysis in Fig. 4.

	Tm [at.%]	Ni [at.%]	Sb [at.%]
20 $^{\circ }$C			
1.1	34.4(7)	34.64(7)	31.0(6)
3.1	41.2(8)	29.9(6)	28.9(6)
3.2	41.8(8)	30.3(6)	27.9(6)
3.3	40.9(8)	30.6(6)	28.5(6)
4.1	10.5(2)	48.9(10)	40.6(8)
4.2	17.8(4)	42.4(8)	39.8(8)
5.1	96.4(19)	2.5(1)	1.1(1)

250 $^{\circ }$C			
1.1	34.9(7)	32.6(7)	32.5(7)
2.1	39.4(8)	23.5(5)	37.1(7)
4.1	2.4(1)	51.5(10)	46.1(9)
4.2	4.2(1)	49.9(10)	45.9(9)
4.3	2.8(1)	51.2(10)	46.0(9)
5.1	68.0(14)	16.6(3)	15.4(3)

500 $^{\circ }$C			
1.1	34.5(7)	31.8(6)	33.7(7)
1.2	35.1(7)	31.7(6)	33.2(6)
2.1	39.2(8)	23.9(5)	36.9(7)
3.1	47.3(9)	26.3(5)	26.4(9)
4.1	7.4(1)	46.2(9)	46.4(9)
4.2	16.0(3)	42.4(8)	41.6(8)
5.1	89.9(18)	5.9(1)	4.2(1)
5.2	86.5(17)	7.7(2)	5.8(1)

750 $^{\circ }$C			
1.1	34.7(7)	32.6(7)	32.7(7)
1.2	35.5(7)	34.3(7)	30.2(6)
3.1	37.1(7)	33.5(7)	29.4(6)
3.2	38.8(8)	31.3(6)	29.9(6)
4.1	3.5(1)	50.9(10)	45.6(9)
4.2	5.7(1)	49.8(10)	44.5(9)
6.1	28.3(6)	36.9(7)	34.8(7)
6.2	20.8(4)	40.2(8)	39.0(8)

1000 $^{\circ }$C			
2.1	39.8(8)	22.8(5)	37.4(7)
2.2	39.0(8)	24.9(5)	36.1(7)
2.3	36.8(7)	28.8(6)	34.4(7)
2.4	38.1(8)	26.4(5)	35.5(7)
3.1	38.0(8)	30.6(6)	31.4(6)
3.2	43.4(9)	28.8(6)	27.8(6)
3.3	37.9(8)	29.3(6)	32.8(7)
6.1	24.2(5)	68.3(14)	7.5(2)
6.2	21.2(4)	45.5(9)	33.3(7)

To the best of the authors knowledge, TmNiSb is the first half-Heusler compound in which PS is intrinsic for ternary composition. Similar formation energies for TmNiSb, TmNi$_{1-x}$Sb and Tm(NiSb)$_{1-y}$ phases under enhanced pressure are the most likely reason for appearance of such peculiar microstructure. Three predicted polymorphic modifications of TmNiSb (cubic, hexagonal and the hypothetical orthorhombic) make obvious association with three types of observed stoichiometry, however no clear quantitative connection was found between those. Further ab-initio study seems necessary to clarify the driving force of this intriguing type of phase separation. PS was not observed for TmNiSb samples prepared at ambient pressures^48,60^, neither it was reported for Sc$_{1-x}$Tm$_{x}$NiSb solid solutions prepared by HPHT method^59^. In the latter case, reason for overlooking this feature was high magnification in EDS mapping (scale bar 1 $\upmu$m), allowing only observation of elemental distribution in single grain and possibly the neighboring impurity.

Areas marked as (4.X), (5.X), and (6.X) in Fig. 4 correspond to NiSb, pure Tm (or Tm$_2$O$_2$), and other nickel-rich precipitation, respectively. Small amount of Tm- and Ni-rich alloys are distributed randomly, while NiSb seem to be located mainly at the grain boundaries.

### Electron back-scattered diffraction

**Figure 5:**
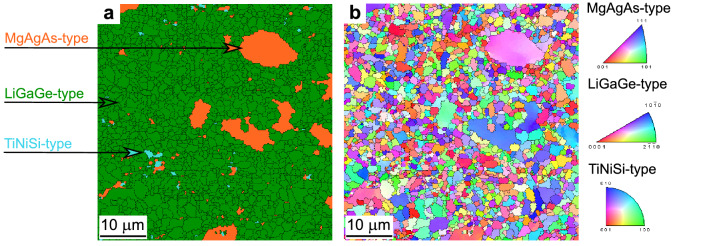
(**a**) EBSD overlay phase map of TmNiSb specimen synthesized at 1000 $^{\circ }$C. Orange, green and cyan regions correspond to MgAgAs-type, LiGaGe-type and TiNiSi-type phases, respectively. (**b**) Inverse Pole Figure (IPF) map in [001] direction and with the IPF color key on the right side.

To confirm proper interpretation of phase transition in TmNiSb synthesized at 1000 $^{\circ }$C, we performed for this sample crystallographic analysis with electron-backscattered diffraction (EBSD) technique. The method enables multiphase crystallographic orientation scans and provides detailed information on each individual component, not only as the phase distribution or grain size, but also the individual misorientations of grains as well as boundaries for each phase separately. In our paper, experimental Kikuchi patterns have been indexed for the MgAgAs-type, LiGaGe-type and TiNiSi-type crystal structures. For cubic and hexagonal phases, lattice parameters were taken from Rietveld analysis, while for orthorhombic phase, which was present in too small amount in the sample (if any) for the refinement, we employed the lattice parameters and atomic coordinates from ab-initio structural relaxation calculations: *a* = 6.783 Å, *b* = 4.392 Å, *c* = 7.498 Å , Tm (0.975; 1/4; 0.603), Ni (0.126; 1/4; 0.071), Sb (0.323; 1/4; 0.312), see below. Panel (a) of Fig. 5 shows overlay fit of phase map. Consistently with XRD analysis, majority (89.2 %) of the examined surface was ascribed to LiGaGe-type phase (green area), while 10.1 % and 0.7 % of the area were determined as the MgAgAs-type (orange) and TiNiSi-type (cyan) phases, respectively. The cubic phase formed a relatively large, irregular and well-separated grains. Presence of large grains of low temperature (MgAgAs-type) phase indicates on the fact, that structural transition was not complete in our sample. It is also necessary to underline than minuscule amount of TiNiSi-type phase detected still does not allow for unambigous confirmation of its real presence in sample studied. Around 2-3 % of the area were difficult to index during the preliminary analysis. The effect could be ascribed to minuscule amount of impurity phases, e.g. NiSb, or Tm$_2$O$_3$, see “Scanning electron microscopy, energy dispersive X-ray spectroscopy” section. Random distribution of the impurities led to their automatic incorporation to grain boundaries or neighboring phases in the final analysis. Minuscule content of the contaminant is expected to have minor influence on the most important conclusions from EBSD.

The middle and right panels of Fig. 5 shows the inverse pole figure (IPF). IPFs are used to present the grain orientation distribution, where each color is related to the different crystallographic orientation. As apparent from the figure, orientation of the grains of LiGaGe-type phase are independent from large MgAgAs-type areas. This feature is indicative of reconstructive phase transitions, i.e. transformations between structures that do not share group-subgroup relations. Transitions of that kind require breaking of near-neighbor bonds, strong change in coordination numbers and structural patterns, which is indeed observed for TmNiSb, c.f. Fig. 1. Similar situation was observed by EBSD for TiPtGe, which exhibits structural transition between MgAgAs-type and TiNiSi-type unit cells^7^. Speculatively, we assume that the structural path of TmNiSb under pressure is starting from low temperature polymorph in MgAgAs-type, then transfers through metastable LiGaGe structure and finishes up in orthorhombic TiNiSi-type. Higher temperatures, pressures or longer time of reactions technically unavailable in our equipment might be necessary for stabilization of TmNiSb in TiNiSi-type cell.

### Ab-initio calculations

To support our findings regarding changes in crystal structure of TmNiSb under pressure, we performed series of ab-initio calculations. First of all, we checked whether minimum in total energy for lattice parameters is close to the experiments results. The optimized parameters are *a* = 6.290 Å  for HH compound, *a* = 4.394 Å, *c* = 7.178 Å for LiGaGe-type phase and *a* = 7.123 Å, *b* = 4.417 Å, and *c* = 7.534 Å for TiNiSi-type structures, respectively. The above results support our finding from Rietveld refinement of the HH and LiGaGe-type phase, see “Powder X-ray diffraction” section; slight overestimation (approx. 1 %) of theoretical lattice parameters with respect to the experiment data is typical for DFT methods.

**Figure 6:**
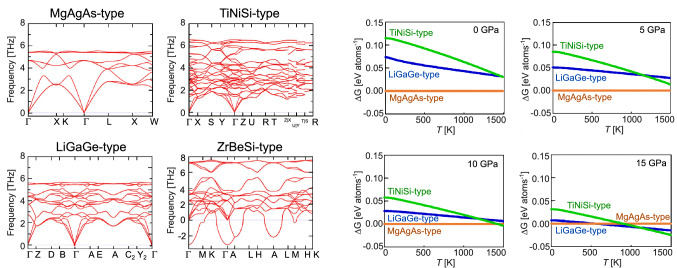
Left panel: phonon dispersion relations for MgAgAs-type, TiNiSi-type, LiGaGe-type, and ZrBeSi-type polymorphic modifications of TmNiSb; Right panel: Temperature variations of Gibbs energy for constant pressures 0–15 GPa.

As the second step of theoretical stability investigation, we calculated phonon dispersions relations for MgAgAs-type, ZrBeSi-type, LiGaGe-type, and TiNiSi-type structures to check thermodynamical stabilities; see left panel of Fig. 6. MgAgAs-type, LiGaGe-type, and TiNiSi-type polymorphs were found to exhibit no imaginary modes, indicating these phases are thermodynamically stable. On the other hand, the hexagonal phase in ZrBeSi-type structure exhibit imaginary modes in $\Gamma$ and *A* points, which suggests, that the structure is thermodynamically unstable. We found Ni and Sb atoms are vibrating very strongly along *c*-axis according to the imaginary vibrational modes. As we mentioned above, Ni and Sb atoms constitute planar structure parallel to *a*-*b* plane, fixed in the *c*-axis direction in ZrBeSi-type. Thus, after relaxing atomic positions of Ni and Sb atoms in the *c*-axis direction with reducing symmetry to *P*3*m* in ZrBeSi-like structure, the imaginary modes disappeared. These atoms eventually formed distorted planar structure in LiGaGe-type, in which imaginary modes were not found. Hence, ab-initio calculations provide additional proof that high temperature high-pressure modification of TmNiSb is in LiGaGe-type structure. We found no further extraordinary features in phonon dispersions like rattling modes or avoided crossing.

The last step in theoretical verification was Gibbs energy calculations in different temperature and pressures (Fig. 6, right panel). The method can provide insight into mechanisms behind structural phase transition. In 0 GPa, MgAgAs-type half-Heusler phase is the most stable up to 1500 K, consistently with current state of experimental knowledge^60^. With increasing pressure, lower-symmetry polymorphic modifications of TmNiSb are becoming closer in energy to HH phase, and eventually in 15 GPa the LiGaGe-type phase is the most energetically stable in range 600–1200 K. Above that temperature, the TiNiSi-type modification attains the lowest energy. This result qualitatively corroborates general understanding of phase transition mechanism—from HH compound through metastable LiGaGe-type phase, up to hypothetical TiNiSi-type polymorph that plausibly can be observed at even higher temperatures and pressures. The interpretation is analogous to the case of TiPtGe^7^. Similar qualitative agreement between theory and experiment, where phase transition was calculated to occur in larger pressures than it was observed experimentally was reported for other HH phases, e.g. for CaAuBi and VCoSb^37,38,42^

### Transport properties

**Figure 7:**
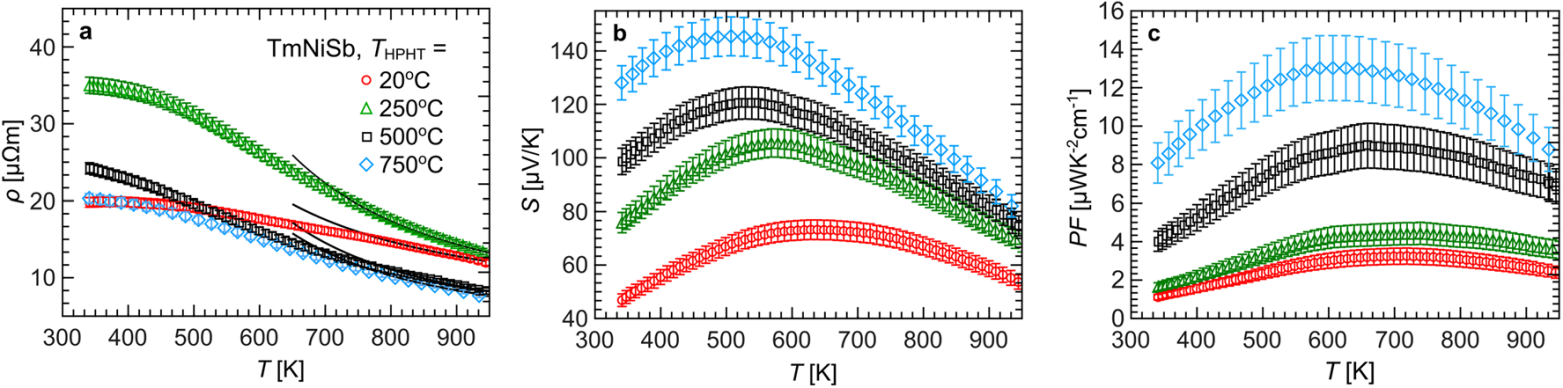
Temperature variations of (**a**) electrical resistivity, (**b**) Seebeck coefficient, and (**c**) power factor for TmNiSb samples sintered at different temperatures.

Electrical resistivity ($\rho$) measurements are shown on Fig. 7a. Values of $\rho$ are moderate, from 20 to 35 $\upmu \Omega$m near room temperature to 8-15 $\upmu \Omega$m at 950 K. The decrease of $\rho$ values with temperature is associated with semiconducting-like charge transfer. In the intrinsic region, i.e. above 750 K, $\rho$(*T*) dependencies were fitted with Arrhenius formula:

1 $$\begin{aligned} \rho (T)=\left[ \sigma _0~exp\left( \frac{-E_{g}}{2k_b T}\right) \right] ^{-1}, \end{aligned}$$

where $E_g$ corresponds to band gap and $\sigma _0$ denotes semiconducting charge transfer coefficient. Both parameters resultant from least-squares fitting are collected in Table 2. The effective band gap increases with temperature of sintering from 170 meV for sample synthesized at room temperature to *c.a.* 260 meV for specimen synthesized at 500 $^{\circ }$C and 750 $^{\circ }$C. Based on the fact that structural disorder reduce $E_g$ of HH compounds^9,68,69^ increase of $E_g$ with $T_{HPHT}$ might be tentatively associated with reduction of the disorder for samples prepared at higher temperatures. The observed $E_g$ for specimens prepared at 500 $^{\circ }$C and 750 $^{\circ }$C could be considered closer to the intrinsic energy gap of TmNiSb, however it is still smaller than result of ab-initio calculations ($E_g$ = 359 meV)^70^. Further precise studies at atomic arrangement in TmNiSb e.g. by synchrotron radiation diffraction and high resolution microscopy appear as interesting topic. Available literature on TmNiSb provides values of electrical resistivity near room temperature in 13–40 $\upmu \Omega$m range^48,59,60^. The discrepancies likely result from sample-specific disorder in crystal lattice and deviations from ideal stoichiometry, typical for HH compounds^51,71^.

**Table 2:** Electrical resistivity, Seebeck coefficient, carrier concentration, Hall mobility, effective mass from parabolic band model near room temperature, as well as parameters obtained from least-squares fit of Eq. (1) to the resistivity data for TmNiSb specimens prepared at temperatures 20–750 $^{\circ }$C.

TmNiSb	$\rho$	*S*	*n*	$\mu _H$	$\sigma _0$	$E_g$	$m_{eff}$	$\mu _w$
	[$\upmu \Omega$m]	[$\mu$V/K]	[10$^{20}$carrier cm$^{-3}$]	[cm$^2$ V$^{-1}$ s$^{-1}$]	[$\upmu \Omega ^{-1}$m$^{-1}$]	[meV]	[$m_e$]	[cm$^2$ V$^{-1}\,$s$^{-1}$]
20 $^{\circ }$C	20(1)	47(2)	1.87(6)	17.0(9)	0.23(3)	170(7)	0.75(6)	24(2)
250 $^{\circ }$C	35(1)	76(4)	1.38(4)	12.8(6)	0.32(3)	238(6)	1.00(8)	23(2)
500 $^{\circ }$C	24(1)	99(5)	3.6(1)	7.2(4)	0.60(4)	262(8)	2.5(2)	24(2)
750 $^{\circ }$C	20(1)	128(6)	1.87(6)	17.0(9)	0.62(4)	260(10)	2.3(2)	77(6)

Results of thermopower measurements are presented in Fig.7b. Two trends are observed within the series: (1) values of maximum thermopower ($S_{max}$) increase with rising temperature of synthesis; (2) temperature at which $S_{max}$ is attained, moves slightly towards lower values with rising temperature of preparation. Due to complex interplay of metallic impurities acting as carrier injectors, porous microstructure for samples sintered at 20 $^{\circ }$C and 250 $^{\circ }$C, possible modification of electronic structure of non-stoichiometric HH phases, we do not attempt to provide here more quantitative reasoning regarding tendencies in *S*. Literature values of thermopower near room temperature for TmNiSb are in range 60-80 $\upmu$V/K^48,59,60^, while $S_{max}$ for TmNiSb samples measured in high temperature regime are 90 $\upmu$V/K at 600 K^59^ and 140 $\upmu$V/K at 550 K^60^. The discrepancies observed for different TmNiSb samples highlight influence of structural imperfections on electron transport of HH compounds.

Figure 7c displays temperature dependences of power factor. Maximum values are achieved in 600–700 K range and increase with rising $T_{HPHT}$ up to 13 $\upmu$WK$^{-2}$cm$^{-1}$ for sample synthesized at 750 $^{\circ }$C, which shows that higher temperature of sintering up to 750 $^{\circ }$C grants better thermoelectric performance. Obtained $PF_{max}$ is similar to those obtained for the other rare-earth-bearing HH compounds^72–74^. It is worth noting that despite phase separation, *PF* of currently examined samples of TmNiSb shown slightly better thermoelectric performance than their arc-melted counterpart (*PF* = 11 $\upmu$WK$^{-2}$cm$^{-1}$)^49^.

Approximate carrier concentration (*n*) and mobility ($\mu _H$) calculated from Hall in single band approximation at 300 K are gathered in Table 2. Values of *n* are moderate, 1.4-3.6 $\times$ 10$^{20}$carriers cm$^{-3}$, similarly to reported previously undoped sample of TmNiSb ($n_H$ = 1.9 $\times$ 10$^{20}$ carriers cm$^{-3}$)^48^ and lanthanide-based *RE*NiSb phases^49^. Mobility attains maximum of 17 cm$^2$ V$^{-1}$ s$^{-1}$ in the studied series. The related HH phase ErPdSb show significantly higher mobility ($\mu _H$ = 165 cm$^2$ V$^{-1}$ s$^{-1}$). The discrepancy might result from different electronic structures of Ni- and Pd- based HH series and distinct microstructure.

Accepting rough approximation of parabolic band modelling for our system, we calculated average effective masses ($m_{eff}$) of TmNiSb samples near room temperature^11^. The results of the calculations together with the values of experimental transport parameters near 300 K are gathered in Table 2. Effective masses are rather low, not exceeding 2.5 $m_e$ for TmNiSb pressed at 500 $^{\circ }$C. Similar value of $m_{eff}$ for TmNiSb was obtained in samples synthesized by arc-melting and spark plasma sintering (2.61 $m_e$)^51^. Last, but not least, we calculated weighted mobility ($\mu _w$) at 340 K from $\rho$ and *S* data using formula proposed in Ref.^75^, see Table 2. The highest value of $\mu _w$ obtained for sample sintered at 750 $^{\circ }$C is consistent with the highest value of $PF_{max}$ observed for this material. The overall higher values of weighted mobility for specimen synthesized at 750 $^{\circ }$C with respect to all other materials, can be further rationalized by the values of Hall mobility and effective mass. In the original form, weighted mobility is $\mu _w = \mu _0 m_{eff}^{3/2}$^76^, where $\mu _0$ denotes the intrinsic mobility, i.e. the upper limit of mobility in pure materials in the intrinsic regime of conductivity. It’s quantitative relation to $\mu _H$ can be found e.g. in Ref.^51^. The difference between $\mu _H$ and $\mu _0$ is usually by factor of smaller than two. In our simplified consideration we might assume, that $\mu _H \approx \mu _0$. Then the product of $\mu _H m_{eff}^{3/2}$ gives values in range 11, 13, and 29 cm$^2$ V$^{-1}$ s$^{-1}$ for samples synthesized in temperatures 20, 250, and 500 $^{\circ }$C. The for TmNiSb obtained at 750 $^{\circ }$C the product of $\mu _H m_{eff}^{3/2}$ gives higher value of 57 cm$^2$ V$^{-1}$ s$^{-1}$, due to good mobility and decent effective mass, which eventually justifies its highest $\mu _w$ obtained with formula from Ref.^75^ and its highest *PF*.

Transport properties of TmNiSb sample sintered at 1000 $^{\circ }$C were not studied due to coexistence of HH and ZrBeSi-type phases, which are likely to make interpretation difficult. High fragility of disc-shaped TmNiSb specimens synthesized by HPHT process made measurements of thermal conductivity ($\kappa$) at high temperatures difficult by standard laser-flash method. Separate project aimed at investigation of PS influence on $\kappa$ will be arranged with related HH phases that show better mechanical properties after HPHT synthesis. Preliminarily, LuNiSb was chosen as the most stable compound from *RE*NiSb series, where *RE* is rare earth metal (unpublished data).

## Conlusions

Samples of *f*-electron half-Heusler compound TmNiSb were synthesized by combination of arc-melting and high-pressure high-temperature sintering in conditions: *p* = 5.5 GPa, *T* = 20-1000 $^{\circ }$C. The pressure-induced phase separation intrinsic for ternary composition was observed by division of HH compound into (1) stoichiometric TmNiSb, (2) nickel-deficient TmNi$_{1-x}$Sb, and (3) thulium-rich Tm(NiSb)$_{1-y}$. For material prepared at 1000 $^{\circ }$C we observed structural transition, result of which was interpreted as hexagonal polymorphic modification with LiGaGe-type structure. Proper interpretation of phase transition is confirmed by refinements in XRD and EBSD experiments as well as phonon dispersion and Gibbs energy energy calculations. Both analogy to known HH counterparts and results of ab-initio analysis suggest possible existence of TiNiSi-type modification of TmNiSb at even higher temperatures and pressures that the currently examined.

Electrical resistivity in 350–950 K range measured for TmNiSb specimens with the phase separation was relatively low, from 5 to 35 $\upmu \Omega$m, and of non-degenerate type. Energy gaps were obtained by Arrhenius fit and led to results of 170–260 meV, where the largest $E_g$ was obtained from specimens sintered at 750 $^{\circ }$C. Seebeck coefficient reached maximum of 145 $\upmu$V K$^{-1}$ at 500 K for sample synthesized at 750 $^{\circ }$C, which resulted in the highest *PF* for this specimen among the series, *PF* = 13 $\upmu$W K$^{-2}$cm K$^{-1}$ at 630 K. Good thermoelectric performance encourages further studied of TmNiSb other HH phases synthesized under pressure. We suggest, that in the future the phenomenon might be used for reduction of phonon transport in half-Heusler thermoelectrics, especially for those having intrinsically high thermal conductivity.

## Methods

Samples were prepared in the first step by arc-melting the elemental constituent (purities Tm 99.9 at.%, Ni 99.999 at.%, Sb 99.999 at.%). For the sake of homogenization, the buttons were remelted and flipped several times. Small amount of Sb from 2 to 5% range was added in order to compensate for the evaporation losses. The obtained specimens were hand-grounded in agate mortar and consolidated at room temperature into a pellet 10 mm in diameter and 2 mm in height at room temperature and 100 MPa pressure. Then the pellet was placed in the special shape container with the graphite heater inside. After applying the 5.5 GPa pressure, the pellet was heated to assumed temperature with the heating rate 16.7 $^{\circ }$C/s. When the specified temperature was reached, the pellet was sintered for 60 s. Then the temperature was lowered to room temperature for 60 s.

Powder X-ray diffraction (XRD) was performed with Xpert Pro PANalytical diffractometer employing Cu K$\alpha$ radiation. XRD patterns were analyzed by Rietveld method incorporated in FullProf software^77^. Density was measured with Archimedes method. Morphology and chemical composition as well as crystallographic orientation and grain boundary type were investigated by using a Field Emission Scanning Electron Microscope (FE-SEM) FEI NovaNano SEM 230 equipped with an energy dispersive X-ray spectrometer (EDS) EDAX Genesis XM4 and a high resolution EBSD camera DigiView V (EDAX Inc. OIM AnalysisTM). SEM images and EDS spectra/maps were performed at 5 and 20 kV acceleration voltage, respectively. Moreover, SEM images were recorded in a beam deceleration mode in order to enhance visibility of morphological features. Since, the surface of the TmNiSb sample sintered at 1000 $^{\circ }$C was too rough and defected to achieve high quality images of Kikuchi patterns (EBSD), the sample was polished prior to scanning. In the first step, the sample was subjected to mechanical polishing using SiC paper from 500 to 4000 grain and SiO$_2$ suspension with particle size equal to 0.05 $\upmu$m (G &G Surface Technology, Italy) to a total removal the surface deformation layers. After that, the sample was cleaned for several times in an ethanol ultrasonic bath and drying using an infrared lamp. Finally, the polished sample was analysed in the scanning electron microscope (SEM) equipped with a TSL OIM analysis software. The Kikuchi patterns were generated at 30 kV acceleration voltage and 32 nA beam current. To produce a crystallographic orientation map, the electron beam was scanned over a selected surface area and the resulting Kikuchi patterns were indexed and analysed automatically (i.e., the Kikuchi bands were detected by means of the software). An image quality (IQ) parameter and a confidence index (CI) were recorded for each Kikuchi pattern. Based on the analysis of the recorded CI value, a multi-phase analysis was realised.

The phonon frequencies and corresponding thermodynamic properties were calculated under the harmonic approximation by the finite displacement method^78,79^ and supercell as implemented in the PHONOPY code^80^. To calculate the total energy of the supercells including displacements, we employed the plane-wave projector augmented wave (PAW) method^81^ in the framework of DFT within the generalized gradient approximation (GGA) functional in the Perdew–Burke–Ernzerhof (PBE) parameterization^82^, as implemented in the Quantum ESPRESSO code^83^. We examined the initial structures of MgAgAs-type, ZrBeSi-type, LiGaGe-type, and TiNiSi-type of TmNiSb. A 0.13 eV smearing width of the Methfessel–Paxton scheme^84^ was used for all crystal structures except for MgAgAs-type TmNiSb. Force constants were obtained from the cells whose sizes corresponded to the 2$\times$2$\times$2, 2$\times$2$\times$1, 2$\times$2$\times$1, and 2$\times$2$\times$1 supercells of the primitive MgAgAs-type, ZrBeSi-type, LiGaGe-type, and TiNiSi-type unit cells, respectively. An atomic displacement of 0.01 Å was used for all supercells. The phonon band paths were determined by using SeeK-path^85^. We used the quasi-harmonic approximation that transforms thermodynamic parameters from the function of volume (*V*) to the function of pressure (*P*)^86^. The Gibbs free energy (*G*) at the finite *P* and *T* was obtained as *G*(*P*, *T*) = min$_V$[*U*$_{\text{el}}$ +*U*$_{\text{ph}}$ + *PV* - *TS*$_{\text{vib}}$], where *U*$_{\text{el}}$, *U*$_{\text{ph}}$, and *S*$_{\text{vib}}$ are the electronic internal energy at 0 K, the phonon energy, and vibrational entropy, respectively.

Hall effect was investigated in PPMS system produced by Quantum Design using perpendicular 4-point method with horizontal rotator. The electrical contacts were prepared with 50 $\upmu$m silver wires and Ag epoxy. Electrical resistivity and Seebeck coefficient in the high-temperature range were measured on commercial Linseis LSR-3 device. To ensure repeatability, for each sample several measurements were made with a different value of the gradient, i.e. 30, 40 and 50 K; in the article results with 50 K gradient are shown. Experimental uncertainties were 3% and 5% for $\rho$ and *S*, respectively.

## Supplementary Information

Supplementary Information.

## Data Availability

The data set relevant for the current study is available from the corresponding author on reasonable request.
