# Supplementary material for: Half-Heusler phase TmNiSb under pressure: intrinsic phase separation, thermoelectric performance and structural transition
Source: Sci Rep. 2023 Jan 28;13:1592. doi: 10.1038/s41598-023-28110-4 (PMC9884295; doi:10.1038/s41598-023-28110-4)
Supplement: Supplementary file 1 — Supplementary Information. [file 41598_2023_28110_MOESM1_ESM.pdf]

Supplementary Information for "Half-Heusler phase TmNiSb under pressure: intrinsic phase separation, structural transition and thermoelectric performance"

Kamil Ciesielski[1,*], Karol Synoradzki[1,2], Damian Szymański[1], Kazuki Tobita[3], Katarzyna Berent[4], Patryk Obstarczyk[1], Kaoru Kimura[3], and Dariusz Kaczorowski[1, 5]

[1] *Institute of Low Temperature and Structure Research, Polish Academy of Sciences, ul. Okólna 2, 50-420 Wrocław, Poland*
[2] *Institute of Molecular Physics, Polish Academy of Sciences, M. Smoluchowskiego 17, 60-179 Poznań, Poland*
[3] *Department of Advanced Materials Science, The University of Tokyo, 5-1-5 Kashiwanoha, Kashiwa, Chiba 277-8561, Japan*
[4] *AGH University of Science and Technology, Academic Centre for Materials and Nanotechnology, al. Mickiewicza 30, 30-059 Kraków, Poland*
[5] *Centre for Advanced Materials and Smart Structures, Polish Academy of Sciences, Wrocław 50-422, Poland*
Email: k.ciesielski@intibs.pl

Tab. S1. Atomic positions for equiatomic ternaries *MTZ* with different crystal structures.

atom	MgAgAs-type	TiNiSi-type	ZrBeSi-type	LiGaGe-type
M	4*b* (½,½, ½)	4*c* (0.02 ± x_1, 0.18 ± y_1, ¼)	2*a* (0, 0, 0)	2*a* (0, 0, z_1)
T	4*c* (¼, ¼, ¼)	4*c* (0.14 ± x_2, 0.56 ± y_2, ¼)	2c (⅓, ⅓, ⅓)	2*b* (⅓, ⅔, z_2)
Z	4*a* (0, 0, 0)	4*c* (0.77 ± x_3, 0.62 ± y_3, ¼)	2*d* (⅔, ⅔, ⅔)	2*b* (⅓, ⅔, z_3)

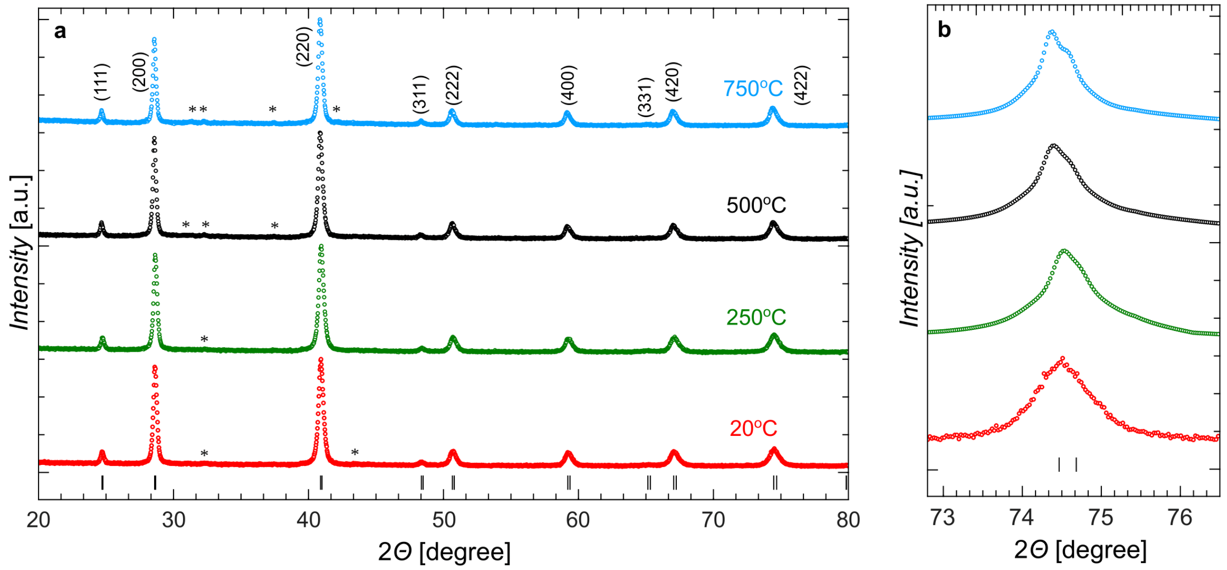

Fig. S1. (a) XRD patterns of TmNiSb samples sintered at temperatures 20-750°C. Ticks at the bottom mark Bragg positions corresponding to the TmNiSb HH MgAgAs-type structure, while asterisks denote maxima from impurity phases. Panel (b) presents closer view on (422) maximum.

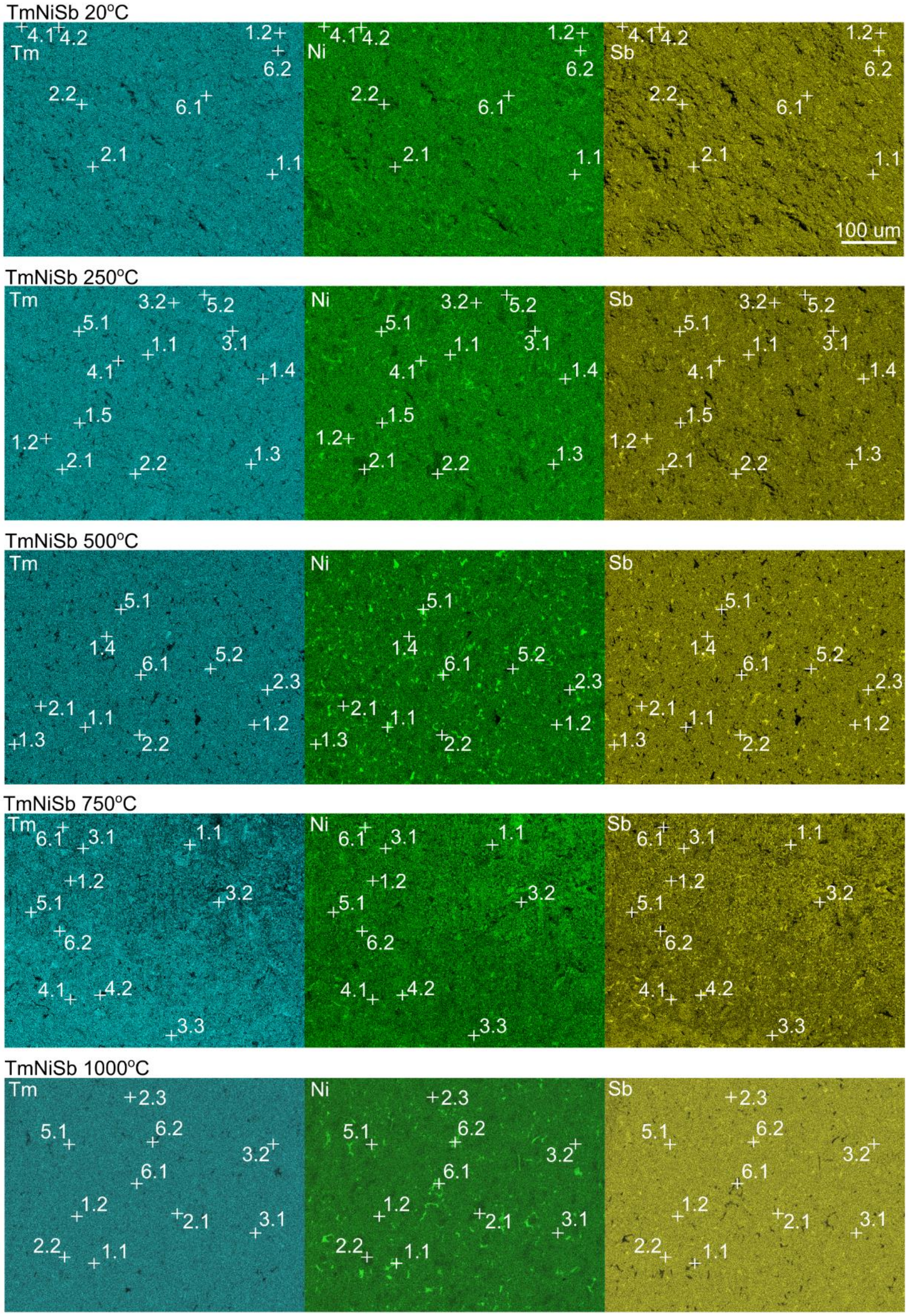

Fig. S2. EDS maps of TmNiSb specimens sintered at T = 20-1000°C. See Tab. S2 for reference of points according to X.Y system, where X denotes captured phase (1- TmNiSb, 2 – $TmNi_{1-x}Sb$, 3 – $Tm(NiSb)_{1-y}$, 4 – NiSb, 5 – Tm, 6 – Ni rich precipitation), and Y is the ordinal number of particular point.

Tab. S2. Results of EDS analysis in points enumerated in Fig. S2.

**20ºC**	Tm [at.%]	Ni [at.%]	Sb [at.%]
1.1	35.0(7)	31.8(6)	33.2(7)
1.2	35.9(7)	30.9(6)	33.2(7)
2.1	38.8(8)	24.5(5)	36.7(7)
2.2	40.0(8)	24.0(5)	36.0(7)
4.1	17.2(3)	55.4(11)	27.4(5)
4.1	4.4(1)	52.2(10)	43.4(9)
6.1	20.1(4)	40.6(8)	39.3(8)
6.2	31.7(6)	38.5(7)	29.8(6)
**250ºC**	Tm [at.%]	Ni [at.%]	Sb [at.%]
1.1	33.6(7)	33.2(7)	33.1(7)
1.2	33.4(7)	33.4(7)	33.3(7)
1.3	34.7(7)	33.0(7)	32.3(7)
2.1	39.8(8)	23.2(5)	37.0(7)
2.2	40.3(7)	22.9(4)	36.9(7)
3.1	35.9(7)	31.2(6)	32.9(7)
3.2	36.8(7)	31.1(6)	32.1(7)
4.1	1.4(1)	52.3(10)	46.3(9)
5.1	60.5(12)	20.7(4)	18.8(4)
5.2	45.3(9)	27.1(5)	27.7(6)
**500ºC**	Tm [at.%]	Ni [at.%]	Sb [at.%]
1.1	35.9(7)	33.6(7)	30.5(6)
1.2	36.3(7)	30.6(7)	33.1(7)
1.3	36.0(7)	32.2(6)	31.8(6)
1.4	36.0(7)	30.7(6)	33.3(7)
2.1	39.2(8)	24.8(5)	36.(7)
2.2	39.8(8)	23.9(5)	36.3(7)
2.3	38.2(8)	26.8(5)	35.0(7)
5.1	55.7(11)	42.6(9)	1.7(1)
5.2	97.1(19)	1.7(1)	1.2(1)
6.1	18.8(4)	75.7(15)	5.5(1)
**750ºC**	Tm [at.%]	Ni [at.%]	Sb [at.%]
1.1	34.4(7)	34.9(7)	30.7(6)
1.2	35.2(7)	35.4(7)	29.4(6)
3.1	38.9(8)	32.3(6)	28.8(6)
3.2	42.0(8)	28.8(6)	29.2(6)
3.3	37.9(8)	30.8(6)	31.3(6)
4.1	11.6(2)	44.9(9)	43.5(9)
4.2	4.2(1)	49.7(10)	46.1(9)
5.1	66.9(14)	28.1(6)	5.0(1)
6.1	43.1(9)	54.7(11)	2.2(1)
6.2	38.2(8)	42.9(9)	18.9(4)
**1000ºC**	Tm [at.%]	Ni [at.%]	Sb [at.%]
1.1	34.0(7)	36.4(7)	29.6(6)
1.2	36.0(7)	32.4(7)	31.6(6)
2.1	39.7(8)	23.6(5)	36.7(7)
2.2	40.3(8)	22.5(5)	37.2(7)
3.1	36.7(7)	31.7(6)	31.6(7)
3.2	35.4(7)	32.2(6)	32.4(6)
5.1	42.2(8)	27.5(6)	30.3(6)
6.1	29.1(6)	52.7(11)	18.2(4)
6.2	17.5(4)	50.1(10)	32.4(7)
